# Persistent Hypercalcemia: Diagnostic Complexity With Multiglandular Hyperparathyroidism, Renal Cell Carcinoma, and Hereditary Tumor Features

**DOI:** 10.7759/cureus.100616

**Published:** 2026-01-02

**Authors:** Taylor Yuska, McKenzie Clark, Yarden Goldman Gollan

**Affiliations:** 1 Osteopathic Medicine, Nova Southeastern University Dr. Kiran C. Patel College of Osteopathic Medicine, Clearwater, USA; 2 Osteopathic Medicine, Kansas City University College of Osteopathic Medicine, Kansas City, USA; 3 Internal Medicine, University of Miami, Miami, USA

**Keywords:** hereditary tumor syndrome, leser-trélat sign, milk alkali syndrome, paraneoplastic syndromes, persistent hypercalcemia, primary hyperparathyroidism, renal cell carcinoma, thiazide diuretics

## Abstract

Persistent hypercalcemia after parathyroidectomy is a diagnostic challenge that requires careful evaluation beyond the parathyroid glands. We describe the case of a 76-year-old man with chronic kidney disease, resistant hypertension, and vestibular schwannoma who presented with weakness, constipation, and new, dark, raised lesions across his back. He reported undergoing parathyroidectomy one year earlier for presumed primary hyperparathyroidism, after which his hypercalcemia persisted. On admission, serum calcium was 13.1 mg/dL (reference = 8.5-10.5 mg/dL), and examination revealed numerous seborrheic keratoses across his posterior thoracolumbar region, consistent with the Leser-Trélat sign. A Tc-99m sestamibi scan localized a left inferior parathyroid adenoma. MRI of the abdomen revealed a 4.1 × 3.6 × 3.1 cm enhancing posterior right renal mass consistent with renal cell carcinoma (RCC). Further history revealed long-term thiazide diuretic use and consumption of one gallon of milk every other day. He was treated with intravenous fluids, zoledronic acid, and calcitonin with symptomatic improvement and was referred to a tertiary center for surgical evaluation. Persistent hypercalcemia after parathyroidectomy is most often due to multiglandular disease, but it may coexist with other etiologies. This patient’s concurrent parathyroid adenoma, RCC, thiazide use, and high calcium intake illustrate multifactorial hypercalcemia. Additionally, vestibular schwannoma, cutaneous lesions, and resistant hypertension suggest a possible hereditary tumor syndrome such as multiple endocrine neoplasia type 2A, neurofibromatosis type 2, or von Hippel-Lindau disease. This case highlights the risk of cognitive anchoring when hypercalcemia is attributed to a single pathology. Instead, a broad differential should be maintained, including concurrent malignancy or inherited syndromes. Early recognition of overlapping etiologies is essential to prevent delayed diagnosis or missed malignancy in patients with persistent hypercalcemia.

## Introduction

Persistent hypercalcemia after parathyroidectomy is a clinically challenging problem. Among postoperative etiologies, residual parathyroid tissue or unrecognized multiglandular parathyroid disease accounts for most cases of persistent primary hyperparathyroidism [1,2]. However, other mechanisms such as malignancy, medication effects, and metabolic disorders may also contribute to hypercalcemia [1-3]. Because hypercalcemia can arise from more than one mechanism, recognizing coexisting processes is essential for appropriate evaluation and management.

We report the case of a patient with persistent hypercalcemia after parathyroidectomy who was found to have multiglandular hyperparathyroidism, a suspicious renal mass, and clinical features suggestive of an inherited tumor syndrome. This case illustrates the diagnostic complexity that can occur when multiple potential causes of hypercalcemia coexist.

## Case presentation

A 76-year-old male with a past medical history of chronic hypercalcemia, resistant hypertension, hyperlipidemia, persistent atrial fibrillation, chronic kidney disease stage 3a, prior cardioembolic stroke, Alzheimer’s disease, and right vestibular schwannoma status post-resection presented to the emergency department after outpatient labs demonstrated a serum calcium of 13.1 mg/dL (reference = 8.5-10.5 mg/dL).

He reported that one year earlier, he underwent a parathyroidectomy for presumed primary hyperparathyroidism. The patient had hypercalcemia before surgery, which persisted postoperatively. At that time, a preoperative Tc-99m sestamibi scan did not demonstrate any focal parathyroid uptake, indicating no clearly localizable adenoma. During surgical exploration, the right inferior parathyroid gland appeared enlarged and was excised for presumed adenoma. Pathology demonstrated focally hypercellular parathyroid tissue without a definitive adenoma. Intraoperative parathyroid hormone (PTH) levels failed to normalize.

At this presentation, the patient reported generalized weakness, fatigue, weight loss, constipation, worsening memory, declining visual acuity, bilateral upper extremity tremor, and the recent appearance of multiple pigmented skin lesions on his back. He denied bone pain, flank pain, and hematuria. He reported home systolic blood pressures consistently between 190 and 200 mmHg. He had no tobacco or alcohol use. Family history was notable for hypertension in his mother, and his father’s medical history was unknown. Additional history revealed long-term thiazide diuretic therapy predating parathyroidectomy and consumption of approximately one gallon of milk every two days.

**Figure 1:**
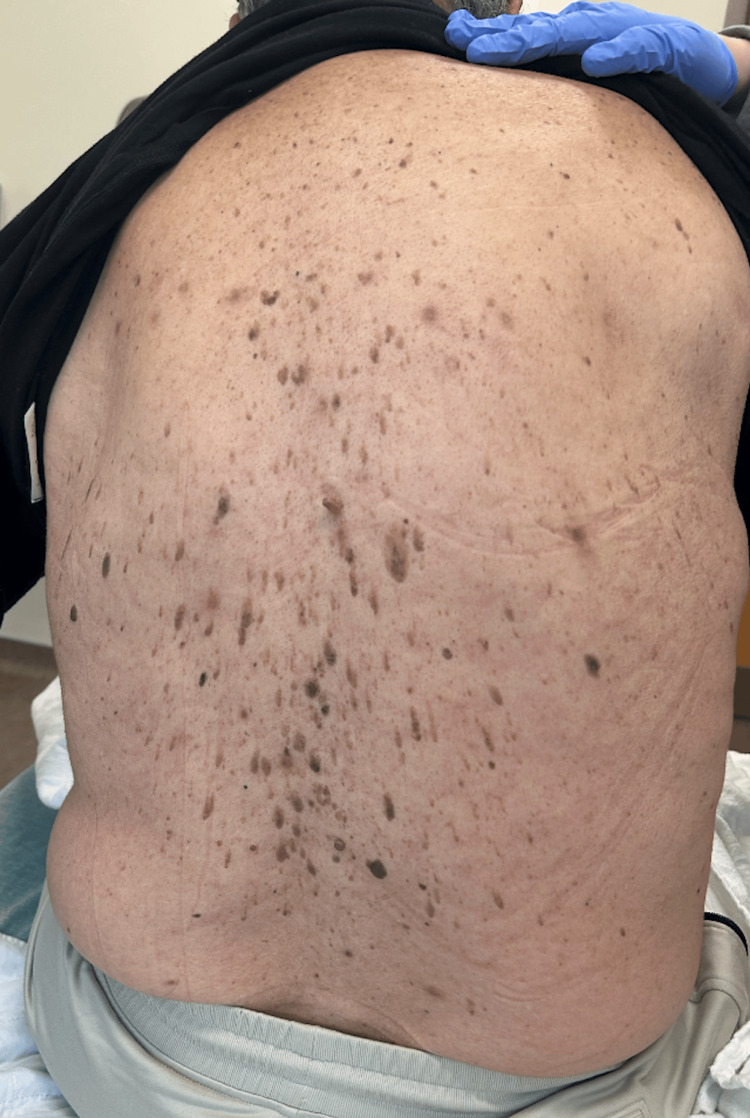
Numerous seborrheic keratoses diffusely across the patient’s posterior thoracolumbar region, resembling the Leser-Trélat sign.

On examination, the patient was alert, oriented, and in no acute distress. Skin examination demonstrated numerous seborrheic keratoses across the posterior thoracolumbar region, resembling the Leser-Trélat sign (Figure 1). The remainder of the examination was unremarkable. Admission labs confirmed hypercalcemia (13.0 mg/dL; reference = 8.5-10.5 mg/dL) with elevated intact PTH (371.9 pg/mL; reference = 15-65 pg/mL), normal vitamin D (31.1 ng/mL; reference = 30-100 ng/mL), and hypophosphatemia (2.0 mg/dL; reference = 2.5-4.5 mg/dL).

Over the first three hospital days, the patient received intravenous fluids, zoledronic acid, and subcutaneous calcitonin, resulting in a decrease in serum calcium to 10.5 mg/dL (reference = 8.5-10.5 mg/dL). Despite oral therapy with irbesartan, carvedilol, amlodipine, and spironolactone, his hypertension persisted with systolic blood pressure ranging from 150 to 170 mmHg.

**Figure 2:**
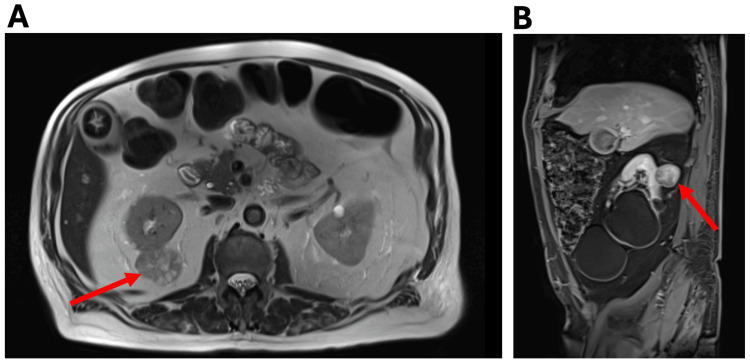
MRI of the abdomen (A and B) revealing an enhancing posterior upper-pole right renal mass measuring 4.1 × 3.6 × 3.1 cm, radiographically consistent with renal cell carcinoma. Image A is a transverse view and image B is a sagittal view. The red arrows indicate the location of the renal mass.

Due to an acute kidney injury, a renal ultrasound was obtained, which demonstrated multiple bilateral renal cysts and a solid vascular hypoechoic lesion measuring 4.6 cm in the right kidney. A subsequent contrast-enhanced CT of the abdomen and pelvis revealed an exophytic solid mass lesion on the right posterior kidney, suggestive of renal cell carcinoma (RCC). The CT also demonstrated bilateral adrenal thickening without features of a discrete adrenal mass. MRI of the kidney confirmed an enhancing posterior upper-pole right renal mass measuring 4.1 × 3.6 × 3.1 cm, radiographically consistent with RCC (Figure 2).

**Figure 3:**
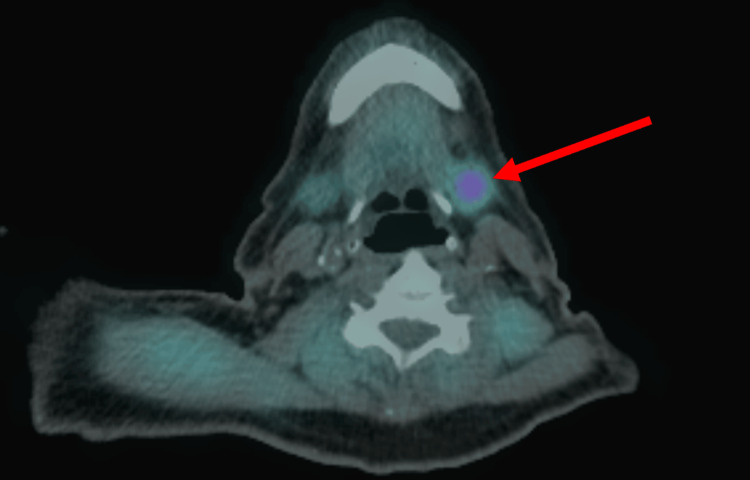
Tc-99m sestamibi scan demonstrating a hyperactive left inferior parathyroid adenoma. The red arrow indicates the location of the adenoma.

A repeat Tc-99m sestamibi scan localized a hyperfunctioning left inferior parathyroid adenoma (Figure 3) and demonstrated radiotracer uptake within the thyroid gland, raising concern for possible thyroiditis. Multidisciplinary consultation was obtained from endocrinology, otolaryngology, and general surgery. Surgical removal of the renal mass was prioritized, and parathyroidectomy was deferred. The patient was referred to a tertiary center for surgical evaluation.

## Discussion

This case illustrates the diagnostic complexity of persistent hypercalcemia after parathyroidectomy, where multiple mechanisms may coexist. Persistent hypercalcemia should prompt a comprehensive reassessment of both parathyroid-dependent and parathyroid-independent causes, as premature cognitive anchoring can result in a missed secondary diagnosis. In this patient, overlapping endocrine, malignant, metabolic, and iatrogenic factors may have acted simultaneously to contribute to his hypercalcemia.

Laboratory findings of elevated calcium, elevated PTH, normal vitamin D, and mild hypophosphatemia initially suggested primary hyperparathyroidism. Persistent hypercalcemia after parathyroidectomy is most commonly due to multiglandular hyperparathyroidism, incomplete resection, or ectopic parathyroid tissue [1]. The elevated PTH and later localization of a contralateral hyperfunctioning gland on Tc-99m sestamibi imaging are consistent with multiglandular hyperparathyroidism. Multiglandular parathyroid disease occurs in 8-33% of cases of sporadic primary hyperparathyroidism and is a common cause of persistent hypercalcemia after parathyroidectomy [1,4]. Though it is important to consider multiglandular disease or ectopic tissue, it is equally important to broaden the differential to include non-parathyroid disease.

The discovery of a right renal mass radiographically consistent with RCC introduced a secondary potential source of hypercalcemia. RCC is an uncommon, but recognized cause of paraneoplastic hypercalcemia, usually mediated by parathyroid hormone-related peptide (PTHrP) secretion [5]. Hypercalcemia has been reported to occur in up to 20-30% of patients with cancer [6]. Although the elevated intact PTH in this case suggests primary hyperparathyroidism, the possibility of concurrent PTHrP secretion cannot be excluded. Concurrent intact PTH and PTHrP secretion has been reported in at least four case reports [7,8]. It is possible that the initial surgical team attributed the persistent hypercalcemia solely to the parathyroid disease, missing the presence of malignancy. This case highlights the importance of avoiding cognitive anchoring and instead maintaining vigilance for concurrent pathologies, particularly malignancy.

Medication effects also may have contributed to the hypercalcemia of this patient. Thiazide diuretics are a well-established cause of hypercalcemia [9]. The mean serum calcium concentration in thiazide-associated hypercalcemia is reported to be 10.7 ± 0.3 mg/dL, with the largest case report being 19.8 mg/dL (reference = 8.5-10.5 mg/dL) [10,11]. It is suggested that the higher frequency of hypercalcemia related to thiazide diuretic use could be due to their action at the sodium-chloride co-transporter in the distal convoluted tubule and increased reabsorption of calcium [9,12]. Potentially, this patient may have had asymptomatic normocalcemic hyperparathyroidism, which became hypercalcemic after starting a thiazide diuretic [13].

Excessive dietary calcium intake likely further compounded this patient’s hypercalcemia. The patient reported drinking a gallon of milk every two days, suggesting a possible element of milk-alkali syndrome. Milk-alkali syndrome is characterized by hypercalcemia, metabolic alkalosis, and renal impairment [14]. This is especially prominent in patients with chronic kidney disease (CKD), as seen in this patient. The presence of CKD and excessive milk consumption further contributes to reduced calcium excretion.

Finally, the coexistence of multiple neoplastic and systemic findings in our patient, including vestibular schwannoma, parathyroid adenoma, thyroiditis, adrenal hyperplasia, pancreatic cyst, resistant hypertension, and cutaneous lesions, also raised the suspicion for underlying hereditary tumor syndromes. Neurofibromatosis type 2 is characterized by bilateral vestibular schwannomas, although unilateral vestibular schwannoma can occur due to genetic mosaicism [15]. Von Hippel-Lindau disease can predispose patients to clear-cell RCC, pancreatic cysts, pheochromocytoma, and, rarely, parathyroid adenomas [16]. Multiple endocrine neoplasia type 2A can cause parathyroid adenoma, medullary thyroid carcinoma, and pheochromocytoma [17]. Although genetic testing was not performed on this patient, the constellation of endocrine and neoplastic findings in this patient is important to consider for appropriate counseling and surveillance.

Limitations

There are several limitations to this case that should be considered. Due to laboratory constraints, measurement of PTHrP was not feasible during hospitalization. Additionally, biopsy of the renal mass was not recommended given its size and radiographical features; therefore, its histology as an RCC was not confirmed. Finally, without genetic testing, the proposed syndromic associations are based on clinical correlation rather than genetic confirmation.

## Conclusions

Persistent hypercalcemia after parathyroidectomy may reflect more than residual parathyroid disease. In this case, primary hyperparathyroidism coexisted with RCC, thiazide-associated calcium retention, excessive calcium intake, and features suggestive of a hereditary tumor syndrome. A comprehensive evaluation with a broad differential is essential to identify coexisting etiologies and prevent delayed diagnosis, particularly of underlying malignancies such as RCC in our patient.
